# Muscle Activation Reduction During Walking with an Active Hip Exoskeleton

**DOI:** 10.3390/biomimetics10010024

**Published:** 2025-01-03

**Authors:** Wentao Sheng, Farzan Ghalichi, Li Ding, Chengtao Yu, Mingyue Lu, Xia Ye

**Affiliations:** 1 School of Mechanical Engineering, Jiangsu University of Technology, Changzhou 213001, China; hitshengwt@163.com (W.S.);; 2 Department of Biomedical Engineering, Sahand University of Technology, Tabriz 51335-1996, Iran

**Keywords:** human–machine interaction, hip exoskeleton, muscle activation, active assist, oxygen consumption

## Abstract

**Objective:** To reduce hip joint muscles’ activation during walking with an active hip exoskeleton. **Background:** Few studies examine the optimal active assistance timing of the hip exoskeleton based on muscle activation characteristics. **Methods:** Sixteen gender-balanced healthy adults (mean age 28.8 years) performed four tasks (each over 20 min). Tasks were different in loading and assistance. Muscle activation was collected by surface electromyography. The collected oxygen consumption evaluated the performance of the proposed active assistance strategy. **Results:** Experimental results verified that lower muscle activation and metabolism could be achieved when the active assistance gait phase was 9–60% of the gait cycle than that of all-gait-cycle active assist. **Conclusions:** Regulating the exoskeleton’s active assistance timing according to muscles’ activation characteristics can improve functional assistance.

## 1. Introduction

Active lower limb exoskeletons have been developed prosperously in this decade and shown great potential in many applications [1,2,3,4,5,6,7]. The applications of these exoskeletons can be divided into three categories: military missions, industrial operations, and healthcare purposes [8,9,10]. Military exoskeletons enhance soldiers’ functions, e.g., physical strength, running speed, leaping ability, and walking endurance [11,12]. Industry exoskeletons assist factory workers in lifting heavy weights or continuous package handling [13]. Healthcare exoskeletons assist patients with walking disabilities [14]. Some healthcare exoskeletons are already commercialized for lower limb rehabilitation and regaining mobility [15,16,17]. These healthcare exoskeletons assist all of the lower limbs’ joints (i.e., hip, knee, and ankle joints) together. The joints’ actuators and frames make these full-body exoskeletons heavy, poorly wearable, and less controllable [6,18,19]. While full-body exoskeletons benefit the safety of human–machine interactions for impaired patients, the potential and benefits of these full-body exoskeletons in assisting mildly impaired patients or healthy subjects are still unclear [20,21].

Single-joint exoskeletons are increasingly emerging to make healthcare exoskeletons more lightweight, portable, and friendly to patients with mild impairment. Ankle exoskeletons are designed to minimize metabolism during walking through optimal assistance timing [22,23]. Knee exoskeletons are designed for human augmentation, such as walking on an inclined surface [24] or regaining normal gait to improve strength and endurance during walking in activities of daily life [25]. Hip exoskeletons are designed to support ambulatory functions in older people [26] or provide lower limb rehabilitation exercises for impaired patients [27]. They can also augment human performance during normal rhythmic walking [28], loaded walking [29], and the manual handling of heavy lifting tasks [30] by providing assistive force/torque. To assist wearers efficiently, assistance magnitude and timing are two critical parameters of the hip exoskeleton’s active assistance strategy. The timing of the hip exoskeleton’s active assistance ensures high performance and safety. There is already abundant research on the optimization of the hip exoskeleton’s assistance magnitude [21]. Therefore, this article focuses on optimizing active assistance timing of the hip exoskeleton.

The state-of-the-art hip exoskeletons’ active assistance strategies mimic the hip joint torque of the human body in the whole gait cycle to carry out the assistance torques in specific proportion [31,32]. However, the lower limb movement of the human body is driven by inertia and weight in some phases during walking, and at this time the muscles do not contract actively but passively. For example, knee extensor muscles passively contract during the swing phase [33]. The metabolism and muscle activation of passive muscle contraction is lower than that of active muscle contraction. The benefits of active assistance in the passive phase of muscle contraction for reducing human energy consumption need further research.

This article proposes an active assistance strategy for hip exoskeletons based on the muscles’ activation characteristics. The hip exoskeleton’s weight is 10 kg. Two force transducers were adopted to calculate and provide feedback on the human–machine interaction torque on hip joints to the exoskeleton’s controller (Raspberry Pi 3B). Firstly, to optimize the exoskeleton’s active assistance timing, hip extensor and flexor muscles’ activation data were collected and analyzed. Hip extensor muscles were chosen as the main assistance muscles. Secondly, the hip exoskeleton’s active assistance timing was optimized (from whole-gait cycle to partial-gait cycle) according to the muscles’ activation characteristics. Thirdly, this article tests the effects of assistance timing on saving walking metabolism and muscle activation. The results highlight that the muscles’ activation characteristics are valuable in optimizing the exoskeleton’s active assistance strategy and illustrate that an optimal assistance timing indeed exists.

## 2. Methods

### 2.1. Data Acquisition and Process

**Figure 1:**
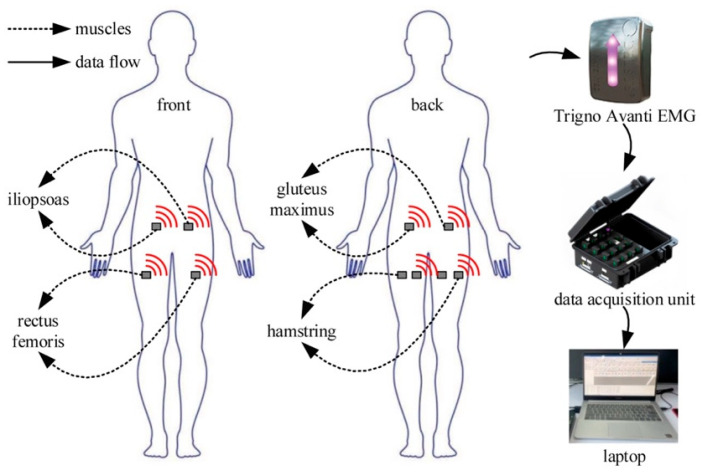
The hardware and data flow in the sEMG collection experiments. There is one sEMG electrode on each iliopsoas, rectus femoris, and gluteus maximus and two sEMG electrodes on each hamstring.

To analyze hip muscles’ activation characteristics, surface electromyography (sEMG) was used to quantify muscle activation in this research. Fifteen healthy subjects were recruited for sEMG acquisition. The demographic information of these subjects is listed in Table 1. All of the experiments’ protocols and details were given to subjects in advance. Subjects all volunteered and gave their consent to the experiments. All human experiments were performed with ethical approval from the Jiangsu University of Technology Ethics board. Informed consent was obtained from each subject. All subjects were asked to walk on a treadmill at a self-selected speed for ten minutes. The hardware and data flow of sEMG data collection experiments are shown in Figure 1. The hip joint’s flexor muscles are mainly the iliopsoas and rectus femoris. The hip joint’s extensor muscles are mainly the gluteus maximus and hamstring [33]. Figure 1 shows eight wireless sEMG electrodes (Trigno Research+ System, Delsys Corp., Boston, MA, USA) attached to the iliopsoas, rectus femoris, gluteus maximus, and hamstring, respectively. The sample frequency of the wireless sEMG signal was set to 1000 Hz.

In the data collection and verification experiments, sEMG data were stored and processed on a laptop (Lenovo, Windows 11, 64-bit, 8 GB RAM, Beijing, China). All data processing codes were edited in Python 3.12. A second-order Butterworth band-pass filter with a 100–400 Hz cut-off frequency was adopted to filter the sEMG signals. sEMG data were processed by root mean square in EMGWorks Acquisition (Delsys Corp., Boston, MA, USA) software. Heel strike (HS) was used to divide sEMG data into gait cycles. Two adjacent HSs were the starting point and the ending point of a gait cycle. The divided sEMG data were time-normalized for further data analysis.

**Figure 2:**
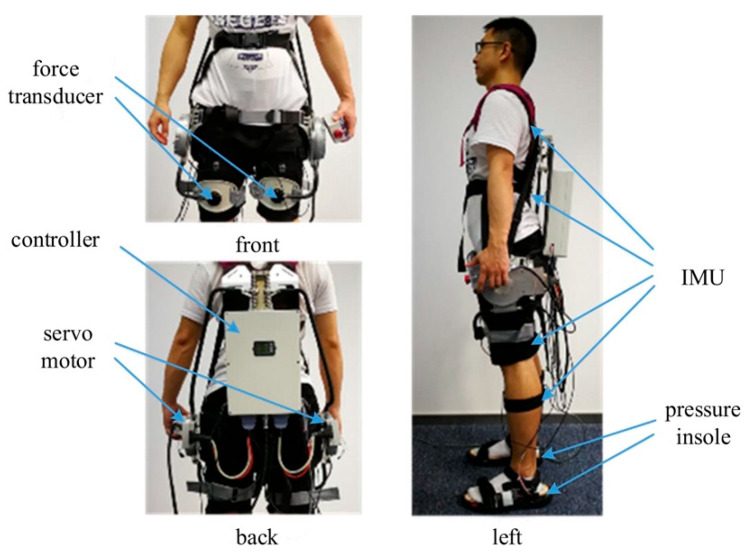
The net weight of the exoskeleton was 10 kg. Six IMUs were attached to the subject’s chest, pelvis, thighs, and shanks to recognize human body gestures. Two pressure insoles were also used to detect HS and TO in real time. There were two force transducers that connect the exoskeleton’s thigh brace with the wearer’s thigh. The transducers were used to calculate and give feedback on human–machine interaction torques for the real-time regulation of assistance torques.

A hip exoskeleton was adopted in the verification experiments. Figure 2 shows two pressure insoles for detecting HS and toe-off (TO). The hip exoskeleton’s weight was 10 kg. Two force transducers were adopted to calculate and provide feedback on human–machine interaction torque on hip joints to the exoskeleton’s controller (Raspberry Pi 3B).

### 2.2. Muscles’ Activation Characteristics

**Figure 3:**
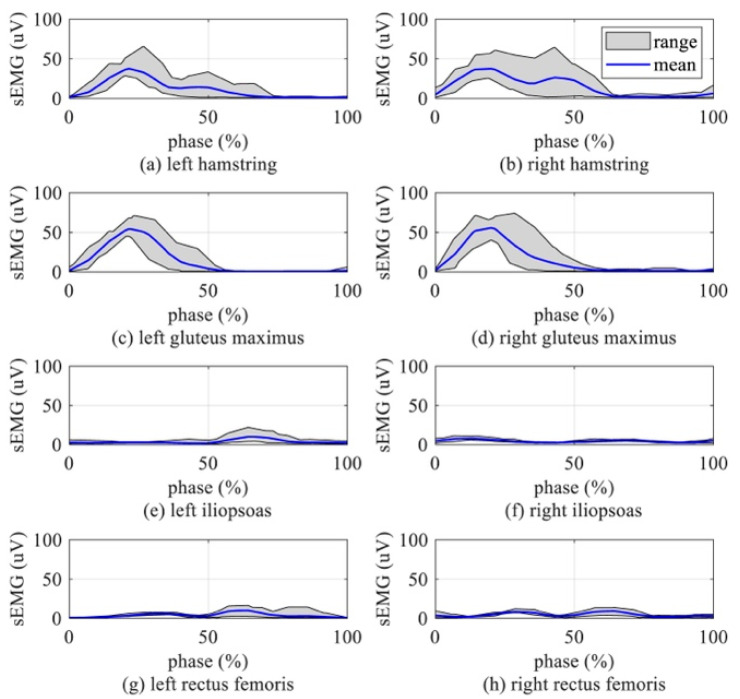
sEMG of left and right hip extensor/flexor muscles during normal walking without exoskeleton. (**a**) sEMG of the left hamstring. (**b**) sEMG of the right hamstring. (**c**) sEMG of the left gluteus maximus. (**d**) sEMG of the right gluteus maximus. (**e**) sEMG of the left iliopsoas. (**f**) sEMG of the right iliopsoas. (**g**) sEMG of the left rectus femoris. (**h**) sEMG of the right rectus femoris.

This section analyzes the activation characteristics of the hip extensor and flexor muscles to optimize the hip exoskeleton’s active assistance timing. sEMG range and mean are shown in Figure 3. The left and right hamstrings’ sEMG range and mean (Figure 3a,b, gray and blue plots respectively) show that the hamstring was activated mainly during 9–60% (stance phase) of a gait cycle. The left and right gluteus maximum sEMG range and mean (Figure 3c,d, gray and blue plots, respectively) show that the gluteus maximus was activated mainly during 9–50% of a gait cycle. The mean sEMG of the hip extensor muscles was larger than 10 uV in the middle of the stance phase, as shown in Figure 3a–d. The hip flexor muscles’ (Figure 3e–h) mean sEMG were all under 10 uV. Hip extensor muscles consumed more energy than hip flexor muscles.

### 2.3. Active Assistance Strategy

**Figure 4:**
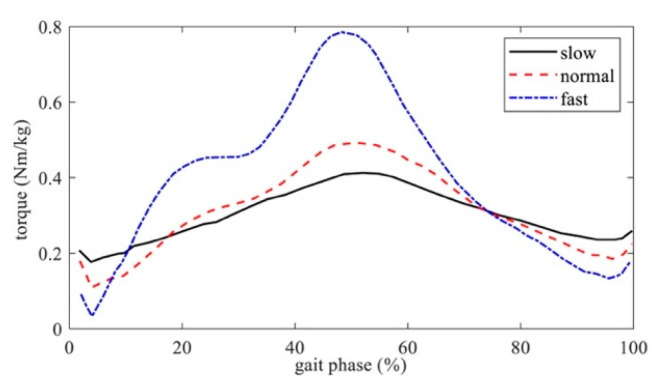
Normalized hip torque in slow, normal, and fast walking [34].

**Figure 5:**
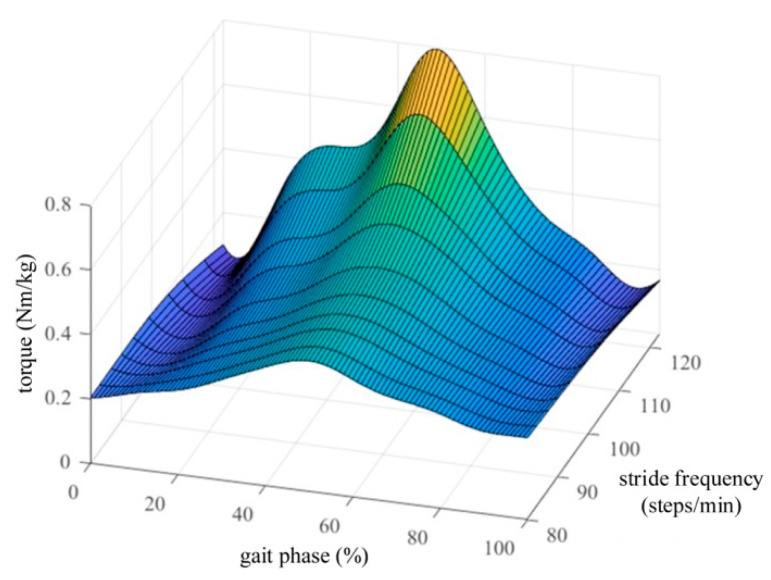
Normalized hip torque surface for 2D-LUT. Normalized hip torque can be obtained according to the gait phase and stride frequency.

Human hip torque online estimation: Human hip torque should be estimated in real time to assist the wearer during walking. Three nominal hip torque curves at different walking speeds (Figure 4, black solid line: 86.8 steps/min; red dashed line: 105.3 steps/min; and blue dot-dash line: 123.1 steps/min) from the Winter database [34] were used to build a normalized hip-torque surface for a two-dimensions look up table (2D-LUT), as shown in Figure 5. The torque surface in Figure 5 was formed by linear interpolation and the surface fitting method. The interpolation intervals in the stride frequency and gait phase dimensions were 5 (steps/min) and 1%, respectively. The 2D-LUT estimates hip torque *T_hip_* (Nm) according to stride frequency *ω_n_* (steps/min) and gait phase *φ_n_* (%), as shown in Figure 5. Stride frequency *ω_n_* is expressed as

(1) $$\omega_{n}=\frac{1}{t_{n}^{HS}-t_{n-1}^{HS}}$$

where *n* is the count of HSs, *n* ∈ *N*^+^, and $t_{n}^{HS}$ is HS time at *n* times; the effective range of *ω_n_* is [80, 125]. *φ_n_* is expressed as

(2) $$\varphi_{n}=\frac{t-t_{n}^{HS}}{t_{n}^{HS}-t_{n-1}^{HS}}\times 100\%$$

where *t* is the present time. Hip torque *T_hip_* is expressed as

(3) $$T_{hip}=T_{hip}^{norm}\times G_{weight}$$

where $T_{hip}^{norm}$ is normalized hip torque estimated by 2D-LUT and *G_weight_* is the weight of wearer. *T_hip_* was used to define tracking values for the closed-loop control of the hip exoskeleton, ensuring that the exoskeleton accurately transmits the required assistance torque to the wearer’s hip joint.

**Figure 6:**
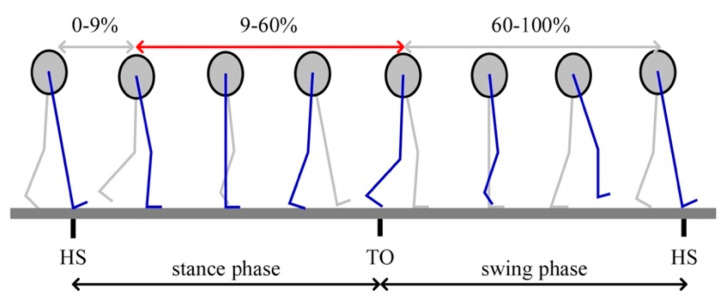
Active assistance timing and lower limb gestures in a gait cycle. The gray double-headed arrow line means passive assistance, and the red double-headed arrow line means active assistance. Active assistance was activated during 9–60% of each gait cycle.

Exoskeleton’s active assistance timing: Hip muscles are activated at a particular phase in a gait cycle, as shown in Figure 3. Hip extensor muscles were chosen as the target muscles to optimize the assistance timing of the exoskeleton. Therefore, the active assistance timing was 9–60% (Figure 6, red double-headed arrow line) when hip extensor muscles are activated in a gait cycle.

**Figure 7:**
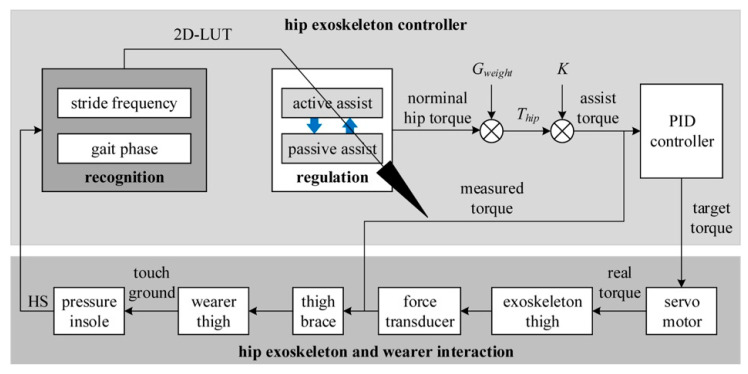
Schematic diagram of hip exoskeleton control framework.

Exoskeleton control framework: An exoskeleton control framework for regulating the exoskeleton’s assistance timing is proposed in this article, as shown in Figure 7. The exoskeleton control framework consists of three modules: (1) Real-time recognition of the wearer’s *ω_n_* and *φ_n_* to estimate the wearer’s hip torque; (2) regulation of active assist and passive assist switching; (3) PID control of the exoskeleton’s joint servo motor. The real-time recognition module calculates the current gait phase and step frequency through the recognized HS timing, and the 2D-LUT regulates the controller to calculate the normalized hip torque. Assistance torque is calculated by multiplying normalized hip torque, subject’s weight *G_weight_*, and assistance proportion *K*. A closed-loop PID control was adopted to accurately carry out assistance torque to the subject’s hip joint. To compensate for the torque caused by the gravity and inertia of the exoskeleton thigh, an accurate feedforward dynamics model was established through the RLS-PSO dynamic parameter identification method [35].

## 3. Experiments and Results

### 3.1. Verification Experiments

**Figure 8:**
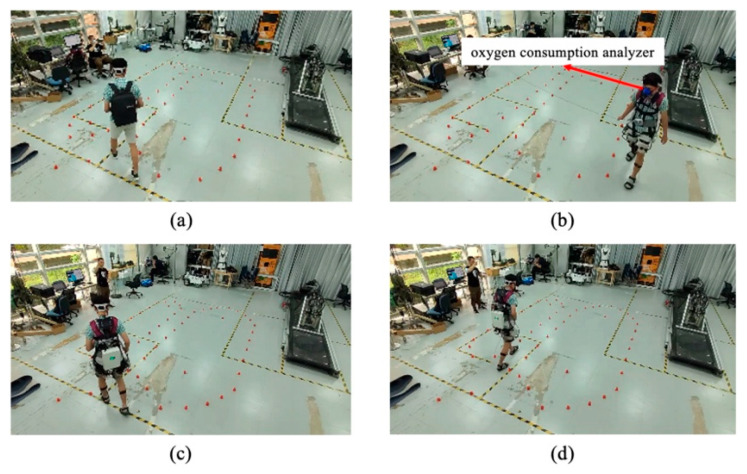
Verification experiments. (**a**) Subjects walked with a 10 kg backpack. (**b**) The exoskeleton only passively assisted the subject. (**c**) The exoskeleton actively assisted the subject during the 9–60% gait cycle phase. (**d**) The exoskeleton actively assisted the subject during the whole gait cycle. Subjects were asked to walk along the pink markers on the ground at self-selected speeds.

Verification experiments were designed to test the effects of assistance timing on saving walking metabolism and muscle activation. The subjects were walking on the ground instead of on a treadmill. There were four settings of the verification experiments, as shown in Figure 8. An oxygen consumption analyzer (K5 4th, COSMED Corp., Rome, Italy) and sEMG electrodes were used to evaluate walking metabolism and muscle activation in all settings. Due to the exoskeleton’s weight of 10 kg, the subjects were asked to walk with a 10 kg backpack when free walking without the exoskeleton, as shown in Figure 8a. As shown in Figure 8b, the hip exoskeleton passively assisted subjects (i.e., there was zero interaction torque between subject and exoskeleton). As shown in Figure 8c, the hip exoskeleton actively assisted subjects during the 9–60% gait cycle phase. As shown in Figure 8d, the hip exoskeleton actively assisted subjects during the gait cycle. The hip exoskeleton’s assistance proportion *K* was set to 0.2. Pink markers were placed on the ground; subjects were asked to walk along the markers at self-selected speeds. Each subject walked along the markers for 10 min; data in the initial and terminal two minutes were not collected to avoid the impact of acceleration and deceleration on walking metabolism and muscle activation.

### 3.2. Experimental Results

**Figure 9:**
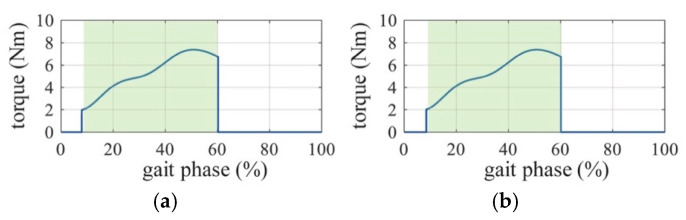
The active assistance timing of the hip exoskeleton in the setting shown in Figure 8c. (**a**) Left side. (**b**) Right side. This is an example from subject 8 during the tenth cycle in the partially active setting. The green rectangles are the active assistance phase planned by the controller. The blue lines are the real active assistance torques executed by the hip exoskeleton.

Accuracy of the exoskeleton’s active assistance timing: The exoskeleton’s active assistance timing should be 9–60% of a gait cycle in the partial active setting. The accuracy of the exoskeleton’s active assistance timing should be tested in advance to verify the effectiveness of the optimal active assistance timing. The accuracy of the bilateral lower limbs was calculated using the method shown in Figure 9. The coincidence degree of the exoskeleton active assistance phase and controller-defined active assistance phase was calculated. The mean coincidence of the partial active setting was 97.68%, which means the exoskeleton accurately executed the controller’s commands.

Walking metabolism: One of the primary purposes of wearing a hip exoskeleton is to reduce the metabolism of human walking. Among the parameters for quantifying human energy loss, metabolism is the most used single index [36]. The measurement of steady-state oxygen uptake (VO2) is considered the gold standard for assessing human metabolism during light or moderate steady-state exercise [37,38]. Thus, VO2 was adopted as one of the indexes that evaluated the performance of the optimized hip exoskeleton active assistance timing.

**Figure 10:**
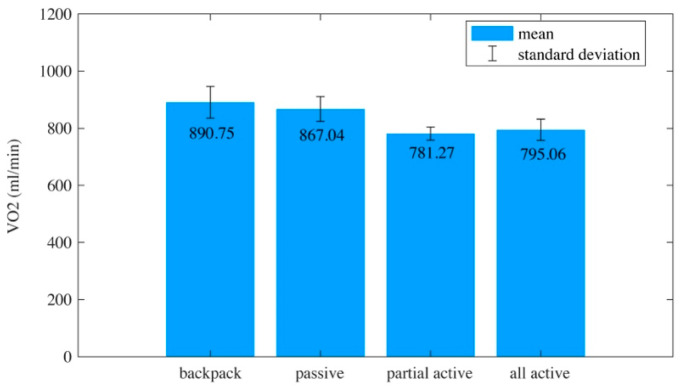
A mean ± std histogram of VO2. The quadratic fit of VO2 computed that the minimum VO2 was achieved with 9.89% reduction from the passive assist mode in the 9–60% active assistance phase.

We used the correlation coefficient *r* and significance test *p* (*α* = 0.05) to analyze which factor affected oxygen uptake. The VO2 in four experiments’ settings are shown in Figure 10. Steady-state VO2 was not eliminated from the results due to it being a constant. The mean VO2 was 890.75 mL/min when subjects walked with a backpack (−0.1 < *r* < 0.1, all *p* > 0.24). The mean VO2 was 867.04 mL/min when the hip exoskeleton passively assisted subjects (−0.1 < *r* < 0.1, all *p* > 0.32). When the hip exoskeleton actively assisted subjects during the 9–60% gait cycle phase, VO2 was 781.27 mL/min (−0.01 < *r* < 0.01, all *p* > 0.27). When the hip exoskeleton actively assisted subjects during the whole gait cycle, VO2 was 795.06 mL/min (−0.1 < *r* < 0.1, all *p* > 0.41). Oxygen uptake was not influenced by variability in the subjects. The quadratic fit (Figure 10, purple curved line) of VO2 computed that the maximum VO2 reduction benefited by 12.29% compared with carrying a 10 kg backpack when the active assistance phase was 9–60%.

**Figure 11:**
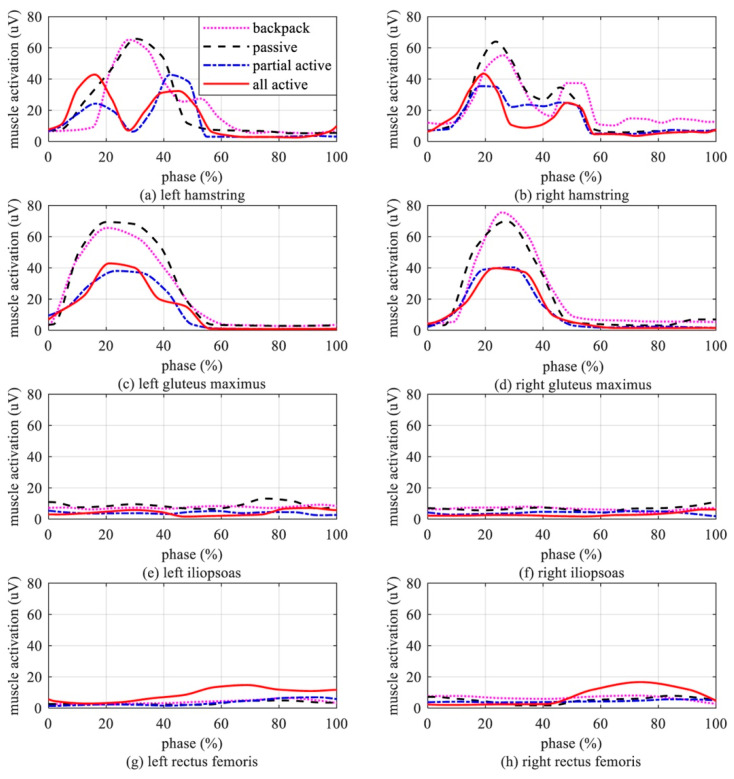
sEMG of left and right hip extensor/flexor muscles. (**a**) sEMG of the left hamstring. (**b**) sEMG of the right hamstring. (**c**) sEMG of the left gluteus maximus. (**d**) sEMG of the right gluteus maximus. (**e**) sEMG of the left iliopsoas. (**f**) sEMG of the right iliopsoas. (**g**) sEMG of the left rectus femoris. (**h**) sEMG of the right rectus femoris.

Hip extensor muscles’ activation: The mean sEMG readings of the hip extensor muscles and flexor muscles are shown in Figure 11. As shown in Figure 11, since the subjects in the experiment carried a 10 kg backpack or wore a 10 kg exoskeleton, the magnitude of the mean sEMG curve was higher than that of the human body without load. However, the waveform pattern of the curve was still highly consistent with that of Figure 3 (*Cc_norm_* > 97% [39]). There was a significant reduction in muscle activation, which can be observed in Figure 11a–d. ANOVA quantified the significant changes in muscle activation. Compared with the passive setting, the magnitudes of the left hamstrings’ activation curves under partial active and all active settings decreased by 35.08 ± 2.3% (*p* < 10^−5^) and 34.84 ± 1.9% (*p* < 10^−6^), respectively. Compared with the passive setting, the magnitudes of the right hamstrings’ activation curves under partial active and all active settings decreased by 44.56 ± 2.2% (*p* < 10^−5^) and 32.11 ± 2.0% (*p* < 10^−6^), respectively. Compared with the passive setting, the activation curve magnitude of the left gluteus maximus under partial active and all active settings decreased by 45.15 ± 2.5% (*p* < 10^−5^) and 38.23 ± 2.2% (*p* < 10^−6^), respectively. Compared with the passive setting, the activation curve magnitude of the right gluteus maximus under partial active and all active settings decreased by 42.4 ± 2.1% (*p* < 10^−5^) and 43.15 ± 1.8% (*p* < 10^−6^), respectively. The activation curve magnitude and mean of the iliopsoas did not change significantly in the four experimental settings, as shown in Figure 11e,f. The activation curve magnitude and mean of the rectus femoris in all active settings were higher than those in the other settings during the 50–100% gait phase. This phenomenon proves that active assistance during the whole gait cycle is not optimal for hip exoskeletons. The sEMG readings of the gluteus maximus and iliopsoas were not obviously changed with or without assistance.

## 4. Discussion

This study aims to explore the impact of the reduction in the assistance timing on the assistance effect of exoskeletons. To exclude the interference of other factors, subjects wore a 10 kg backpack with the same weight as the exoskeleton to conduct walking tests, and this was taken as the control group to prove that optimizing the assistance timing of exoskeletons can reduce sEMG readings and oxygen consumption in the human body under the same load. Overall, the hip exoskeleton with an optimized active assistance timing positively reduced walking metabolism and muscle contraction. The initial hypothesis was accepted: the active assistance of the hip joint in the whole gait cycle did not obtain lower walking metabolism or muscle contraction than that of partial active assistance. The U-shaped trend of VO2 indicates a great potential that an optimal exoskeleton active assistance timing can be obtained through analyzing the activation characteristics of target joints’ muscles.

On the other hand, the increase in metabolism and iliopsoas activation phenomenon in all active settings are consistent with human walking characteristics [33]. Perry demonstrates that the flexion of lower limbs in the swinging phase is a passive movement under the action of inertia in the pre-swing stage, and the flexor muscles are in the passive contraction stage during walking [33]. The increase in the active assistance phase penalizes the metabolism of passive contracting muscles. Thus, assisting magnitudes are not the only concern of exoskeletons in reducing walking metabolism. The exoskeleton’s assistance timing should also be considered as one of the main indexes that benefits walking metabolism reduction.

The findings of this article provide instructive information about the regulation of the exoskeleton’s active assistance strategy. For example, reducing the active assistance duration extends the exoskeleton’s working hours. Therefore, mildly impaired patients can obtain more prolonged periods of exercise rehabilitation without frequently recharging the exoskeleton. Furthermore, wearers need to gradually adapt to the active assistance of the hip exoskeleton in the human–machine interaction. Assistance timing and magnitude affect the efforts and time for the wearer to adapt thoroughly. Therefore, further research in optimizing wearers’ adaptation may be helpful [40].

## 5. Conclusions

This article proposes a hip exoskeleton active assistance strategy optimization method which is more ergonomic. Experimental results verify that analyzing hip muscles’ activation is conducive to optimizing hip exoskeletons’ active assistance timing. Thus, a better performance in reducing walking metabolism and muscle activation is achieved. Furthermore, this article provides new insight into improving single-joint healthcare exoskeletons’ active assistance strategies during walking.

In future works, optimizing the hip exoskeleton’s active assistance timing based on hip muscles’ activation characteristics during circular walking, running, ascending/descending ramps, and ascending/descending stairs will be researched in a larger subject pool.

## 6. Key Points

- A maximum reduction in hip muscle activation was found when the active assistance phase was 9–60% of the gait cycle;
- Surface electromyography and oxygen consumption showed signs of muscle activation reduction using the proposed active assistance strategy;
- Since the findings may point towards applications for activities in the daily lives of elderly people, further research is needed to verify the relation between age and muscle activation.

## Figures and Tables

**Table 1:** Demographic information of subjects.

Subject	Gender	Age (years)	Height (cm)	Weight (kg)
1	Male	25	174.9	68.3
2	Male	28	177.1	75.2
3	Male	22	173.6	62.7
4	Male	31	180.0	71.9
5	Male	28	172.3	55.7
6	Male	24	181.5	78.7
7	Male	35	168.9	57.6
8	Male	33	170.4	61.4
9	Female	27	161.5	49.2
10	Female	21	165.7	51.3
11	Female	20	170.5	54.9
12	Female	28	158.3	47.3
13	Female	32	155.7	48.9
14	Female	35	163.1	52.4
15	Female	34	161.9	61.3
16	Female	37	159.2	65.4
Mean [SD]	-	28.8 [5.4]	168.4 [8.0]	60.1 [9.7]

## Data Availability

The generated and analyzed datasets in the current study are not publicly available due to privacy concerns; however, the anonymized datasets are available from the corresponding author on reasonable request.
